# Wisconsin

**Published:** 1898-05

**Authors:** W. G. Clark

**Affiliations:** Secretary


### WISCONSIN SOCIETY OF VETERINARY GRADUATES.

THE annual meeting was held at Madison, in the rooms of the State Agricultural Society, on Friday, February 25, 1898. The meeting was called to order at 2 o'clock P.M., by the President, Dr. L. A. Wright. Roll-call: Drs. Clark, Leech, Schmitt, and Wright. Visitors: Drs. Clute, Heer, Beattie, and Smith.

The Secretary's report was read and adopted. The Secretary reported correspondence with Dr. J. P. Laws, the Treasurer, who had removed from the State. The Secretary was unable to find the Treasurer's books to investigate the same. The report of the Secretary in regard to the Treasurer's accounts was adopted subject to inspection.

Dr. Clark presented the application of Dr. J. P. Laws for honorary membership. On motion, action on the application was postponed until the Treasurer's accounts were investigated.

Dr. G. Ed. Leech presented an application for honorary membership for Dr. C. H. Ormond, of Milwaukee. It was moved by Dr. Clark, and seconded by Dr. Schmitt, that the application be granted; carried.

Dr. G. Ed. Leech reported that two members of the Society, Drs. R. A. Higgins and John T. Unertl, were holding offices, one as first vice-president, and the other as treasurer of a live-stock insurance company, and were giving the said company free services. Dr. Leech gave a description of the plan of operation of said company and a detailed report of the business transacted by the company during the past year. It was moved and seconded that Drs. John T. Unertl and R. A. Higgins be requested by the Secretary to show cause on or before the next regular meeting in August why they should not be expelled from membership for violation of Section 7 of the Code of Ethics.

The application for membership of Dr. R. S. Heer, Plateville, and Dr. H. P. Clute, Marinette, were presented. In the absence of the Censors, the President appointed Drs. Schmitt, Leech, and Clark to report on the applications. The committee reported favorably on the applications. The President cast a deciding vote, and they were declared elected to membership.

Dr. Leech made a report in regard to the veterinary bill before the last session of the Legislature. After discussion, the report was accepted, and it was decided to present practically the same bill at the next session of the Legislature.

The President gave a short and instructive talk on the duties and future prospects of the veterinarian.

Dr. G. Ed. Leech read a very interesting paper on "Azoturia in the Dog," and reported several cases. Discussed by Drs. Clark, Beattie, Heer, Wright, and Schmitt.

Dr. Wright reported an obstinate case of "Sweeny." Several interesting cases were reported and discussed.

The Society then proceeded to the election of officers for the ensuing year, which resulted as follows: President, Dr. L. A. Wright, Columbus; Vice-President, Dr. J. F. Roub, Monroe; Secretary, Dr. W. G. Clark, Marinette; Treasurer, Dr. Charles Schmitt, Dodgeville; Censors, Drs. R. S. Heer, Plateville; G. Ed. Leech, Milwaukee; and H. P. Clute, Marinette.

It was decided to hold the semi-annual meeting at Columbus in August, subject to the call of the Secretary.

On motion, the Society adjourned.

W. G. Clark, M.D.C.,
Secretary.

